# Lip Reconstruction by Double Layer and Double Flap New Combination Technique: A Case Series

**DOI:** 10.3390/dj10020019

**Published:** 2022-01-27

**Authors:** Antonio Cortese, Salvatore Catalano, Antonio Alberto Maria Giunta, Antonio Borri, Daniele Panetta, Pier Paolo Claudio

**Affiliations:** 1 Unit of Maxillofacial Surgery, Department of Medicine and Surgery, University of Salerno, 84084 Salerno, Italy; antonio-borri@alice.it (A.B.); danielepanetta@gmail.com (D.P.); 2 ENT Department, Santa Maria della Misericordia Hospital, 06121 Perugia, Italy; soter.catalano@gmail.com (S.C.); giunta@gmail.it (A.A.M.G.); 3 National Center Natural Product Research, Department of Biomolecular Sciences, University of Mississippi, Jackson, MS 39216, USA; 4 Department of Maxillofacial Surgery, University of Mississippi Medical Center, Jackson, MS 39216, USA

**Keywords:** lip-reconstruction, plastic surgery, buccinator muscle, orbicularis muscle, oral cancer

## Abstract

In the past, lip reconstruction after ablative surgery has been performed by primary closure and more recently by free flap transfer technique. Cheek’s skin flap has been used to reconstruct the lower lip cutaneous portion. This study presents a reconstructive method for the vermillion and the lip’s cutaneous portion using the Goldstein–Robotti techniques (for the vermillion) and the buccinator flap to reconstruct the cutaneous lip portion and the perioral muscles. This procedure allows a complete reconstruction with a double layer technique for defects of more than one-third of both lips, together or alone, including modiolus, showing satisfactory functionality and aesthetics. The procedure was carried out by splitting the buccinator muscle and elongating the upper and lower buccinator bundles, together or alone. Soft tissue blunt dissection prevented most facial nerves and vessels injuries, ensuring blood supply and an amount of lip sensitivity. Even in the case of facial vessel ligatures after neck dissection, the technique was possible basing the flap pedicle on the internal maxillary artery branches (buccinator) and contralateral facial vessels (orbicularis). We present a case series of six reconstructions of various defects of the upper and lower lips, including the commissure after ablative surgery for squamous cell carcinoma and polymorphous adenocarcinoma. The results showed satisfactory functional and aesthetic outcomes, with similar tissue texture, static and dynamic symmetry achieved for all the patients.

## 1. Introduction

Head and neck cancer is the sixth most common type of cancer globally [1]. Among these neoplasms, squamous cell carcinoma (SCC) of the lower lip includes more than a quarter of all oral cancers [2]. Lip SCC is more frequent in male patients over 45 years of age, especially in those with chronic sun exposure, smoking, and alcohol abuse, along with the concomitance of systemic lupus erythematosus [2,3], cheilitis, leukoplakia: sunray chronic exposure, alcohol, and cigarettes smoking is the most important risk factors alone or in association [3,4,5]. Accumulation in DNA damaging after risk factors exposure alone or in combination with flaws in DNA repair mechanisms play a role in cancer onset and growth. Recent studies to identify predictive DNA repair biomarkers in lip cancer to improve diagnosis, prevention and treatment were carried out [6].

In the literature, several techniques have been described to reconstruct the lips that range from simple primary closure to the use of local or free transfer flaps, each of them with specific advantages and disadvantages. Reconstruction of the lips has ancient roots in the history of facial plastic surgery. All the latest techniques are based on the old Master of Surgery and sometimes are variants or associations of old methods. The most common reconstructive techniques are the so-called “fan flap” according to Gillies, the Estlander lip exchange technique, and the “universal principle” of the lower lip reconstruction according to Grimm.

Following Gillies, the basic principle of lip reconstruction postulates the use of “like with like” tissues, avoiding different skin textures with annexes, thereby reconstructing the resected lip by incision and flap rotation of a lip sector from the opposite side. The technique’s rationale was to obtain a suitable aesthetic and functional reconstruction of the sphincter ring by transferring tissue from the unaffected lip. By these techniques, microstomia will commonly result from dimension reduction of the sphincter ring: in effect, flap tissue was achieved from the opposite unaffected lip, thus transferring the defect from one lip to another lip area without gaining any new tissue.

Because of the heavy impact on face aesthetic and function of ablative surgery and the reconstructing difficulties, other treatment strategies for squamous cell carcinoma (SCC) have been proposed to avoid ablative surgery. An example is electrochemotherapy, which has been used in patients who cannot undergo surgery or are refusing ablative surgery [7,8].

Different reconstructive techniques have been proposed and discussed in the literature to manage difficulties and drawbacks. We believe that suitable anatomic, aesthetic and functional lip reconstruction can be more appropriately achieved with local flaps than other kinds of flaps. To improve local flap results, we have proposed a new method to reconstruct resected lip, combining two different flaps for two different reconstruction levels, gaining tissue from the cheek (by buccinator flap) and sliding the elastic vermilion structures (by orbicularis Robotti flap), to satisfy both the anatomical and the functional need [9].

The clinical history of SCC of the lower lip is aggressive in nature because it can invade the muscle deeply up to the jaw, with bone erosion and invasion in advanced cases: metastases are commonly related to the lymph nodes in the neck.

All of the cases were patients affected by SSC except one who showed a polymorphous adenocarcinoma (PA). Polymorphous adenocarcinoma (PA) is a rare malignant neoplasm that frequently occurs in the minor salivary glands in the palate and oral cavity. Head and neck PAs most frequently occur in the sixth and seventh decades with a female predilection. Localization to the lip is rarely observed (only 7%). The average PA tumor size is about 2 cm at presentation with rare distant metastasis (4.3%). It has an excellent survival outcome, with surgery being the treatment of choice [10].

For SSC cases, radical surgical resection, including tumor excision with 1.5 cm of free margins and neck dissection for lymph nodes removal, is fundamental in the treatment plan [11]. Other treatment options are chemotherapy and radiotherapy alone or in combination and recently electrochemotherapy [7,12].

After excision of the lip, reconstruction includes the primary closure for minor excisions or the reconstruction using free flaps or local flaps. Compared to reconstruction with free flaps, primary closure and local flaps are advantageous for less scar tissue after reconstruction, better function, aesthetic appearance, and less donor site morbidity. Primary closure is a simple surgical method. The scar has a more linear shape but is not suitable for larger excision surgery when more extensive lip cancer results in microstomia. In cases where the excision is more significant, local flaps with various methods such as the Abbe or Estlander flap, the Bernard flap, and the V-Y advancement flap are currently proposed as a better choice for reconstruction [13].

Particularly for total thickness and extensive resections involving different areas of the lips, reconstruction is challenging to restore skin and mucosa shape, structure, function, with similar skin and mucosa texture, colors, and annexes. For these reasons, we consider local flaps more suitable for lip reconstruction than other kinds of flaps.

With the current techniques, it is more challenging to reconstruct lip defects after full-thickness resections exceeding one-third of the lip; moreover, it is more challenging to reconstruct demolitions involving a hemi-lip and the commissure and the opposite lip. Most lip reconstruction methods use flaps that transfer tissues from the upper or lower opposing lip, and therefore, they are not suitable when both the upper and lower lip and the commissure are involved. The basic principle of most popular lip reconstruction techniques, according to Estlander, Gillies, and Millard, consists of repairing the lip by rotation of a sector from the opposite lip to reconstruct the muscular ring. Because of the tissue transferring from the opposite lip, the final amount of tissue for the orbicularis oris ring will be insufficient. Additionally, commissuroplasty to correct the microstomia will result in a lack of muscular orbicularis and interruption with aesthetic and functional drawbacks because of the lack of orbicularis oris tissue gaining and second stage need [14].

The lips have two different functional and aesthetic anatomical structures: the cutaneous area and the vermillion portion with different tissue structures and muscles. The cutaneous portion has a muscular system for lip expansion and mobility, while the orbicularis oris ring is functional to the sphincter function as in suction: combination and overlapping of the two systems are needed for good aesthetic and functional results after reconstruction in speech, face expressivity and mastication.

For these reasons, those techniques that use cutaneous portions from the opposite lip still have poor aesthetic and functional (microstomia) results as they transfer the defect from one lip to another without any significant final tissue gain. Other techniques that use tissues from different areas, such as in regional or distant free flaps, are ineffective because they cannot restore the orbicular ring of the lips in its anatomy, aesthetic function, and tissue texture with annexes.

This paper aimed to show the results of the new procedure we developed combining two different techniques: the Robotti technique for vermillion and orbicularis muscle ring reconstruction and a muco-myo-cutaneous cheek flap based on the buccinator muscle pedicled on the facial or on the internal maxillary vessels for the cutaneous lip portion, thereby correctly restoring the two levels of the lip anatomy and function [9,12].

This technique is particularly effective because the buccinator muscle is the anatomical continuation of the orbicularis muscle before the decussation of the fibers at the modiolus. This feature can be usefully exploited to reconstruct the anatomy of the lower or upper lip or both because the flap tissue is very similar in structure and naturally fit in the defect after lip resection for tumor ablation.

## 2. Materials and Methods

### 2.1. Patients

From September 2016 to March 2021, within the Maxillofacial Reconstructive Surgery Unit, San Giovanni di Dio and Ruggi d’Aragona University Hospitals of Salerno, a series of about 20 patients underwent the reconstruction of lip and commissure defects after ablative surgery. Among this group, we selected 6 cases for chart review who were reconstructed, after ablative surgery, by a combination of composite buccinator cheek flaps following our technique in association with orbicularis muscle and vermillion reconstruction flaps following the Robotti technique (Table 1).

Demographic, clinical, and surgical data were collected through medical records, clinical photographs, and interviews for all patients. Inclusion criteria selected patients affected by malignant tumors candidate to full-thickness resection of the lips. We excluded from the chart review patients who showed distant metastasis, neurological diseases, who had a relapse after previous treatments for the same tumors, patients showing partial-thickness lower lip defect, or lower lip defects with less than 1/3 of the lower lip length and/or incomplete medical records/follow-up.

The selected group consisted of 6 patients, 5 males, and 1 female, with a median age of 64 years. All the selected patients showed a defect, after ablative surgery, involving the lower and/or upper lip for an area larger than 30%, or involving the oral commissure as in all cases, but one. All male patients were diagnosed with squamous cell carcinoma of the lip, while female patients were diagnosed with polymorphous adenocarcinoma, as summarized in Table 1.

All patients were pre-operatively studied by computed tomography scan and echography, confirming the local tumor extension without any lymph nodes involvement also at echography of the neck for lymph nodes hilum check. Patients were admitted to the hospital the day before, and prophylactic intravenous antibiotics were administered. Because of negative signs for lymph nodes involvement at pre-operative evaluation, no neck dissection was planned, only performing careful postoperative follow-up by echography every 3 months for the first two years; and every six months for late follow-up.

Excision was performed full-thickness with a safe margin of 1.5 cm of tissue free from the tumor at clinical evaluation at the surgical field. After excision, fresh biopsies were performed on the margins around the excision resulting in free from tumor invasion.

After excision, the reconstruction was performed in all patients with our technique, in the same surgical stage. The follow-up period ranged from 1 month to over 3 years (average: 24 months, median: 29.5 months). All subjects signed a consent form, following the 1975 Helsinki Declaration, amended in 1983. For this study, approval was obtained from the local ethical council for institutional research (protocol n° 38/06).

### 2.2. Surgical Reconstruction Technique

**Figure A1:**
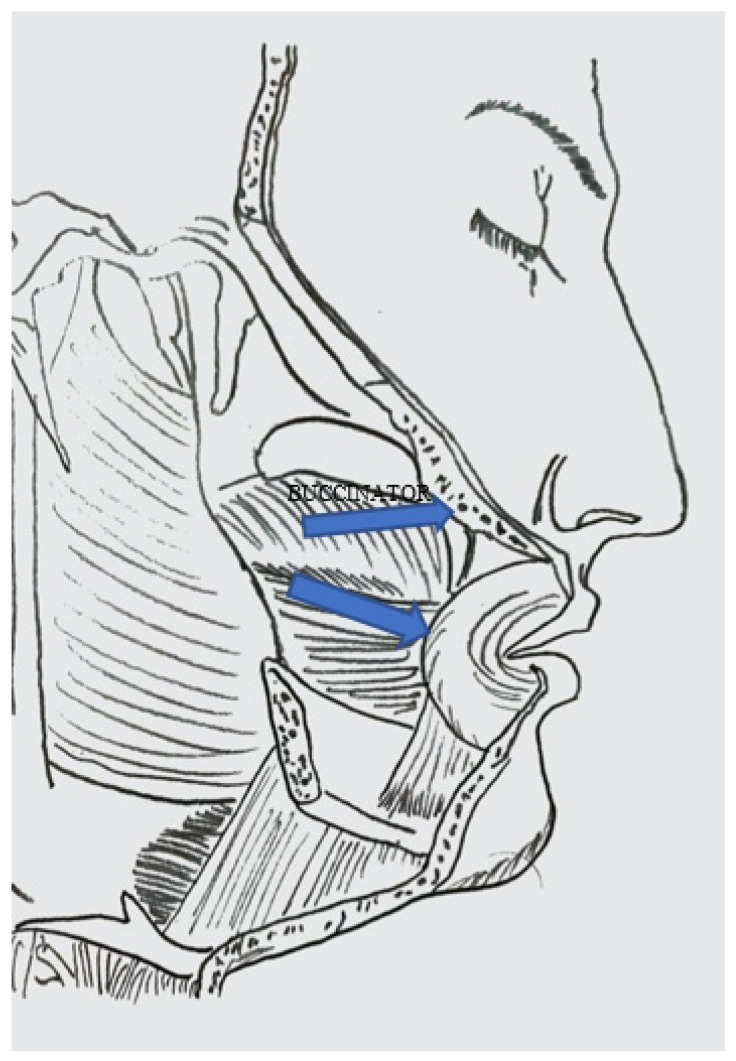
Buccinator flap two bundle.

The anatomy and function of the lip were respected because the transfer took place with careful blunt preparation of the flap by vascularization and most of the innervation, and both areas (cutaneous and vermillion) were reconstructed with two different techniques, one for each lip area. The cutaneous portion was reconstructed through a composite muco-myo-cutaneous cheek flap and the vermillion area by sliding the flap following the Robotti method using the orbicular muscle with vermillion from the contralateral half of the lip remained undamaged. After ablative surgery, reconstruction started from the cutaneous portion of the cheek flap, sculpturing a V-Y flap at the buccinator muscle area. After V-Y skin and mucosa cheek incisions, muscle flap layer preparation started by blunt dissection of the two buccinator muscle bundles, preserving the facial vessels supply and most of the sensitive and motor nerve supply (see Figure A1). The two bundles were divided at the distal end for cutaneous (external area of the lips defect) lip reconstruction (upper, lower, or both lips even with modiolus) together with skin and mucosa layers. In this way, the composite flap’s advancement was possible, preserving facial vessels and most of the sensitive and motor nerve supply. The two bundles with skin and mucosa layers were divided to reconstruct both upper and lower lips for the resected areas. Still, even in the case of upper or lower lip alone demolition, reconstruction was possible by our flap technique using only one bundle. The neo-modiolus was reconstructed by suturing the two bundles of the buccinator muscle at the desired site after V-Y advancement. Skin and mucosa layers of the flap were sutured following the V-Y method. Vermilion area with orbicularis oris muscle ring was reconstructed by the sliding flap following the Robotti technique that was possible because of the elastic properties of the flap [9].

**Figure A2:**
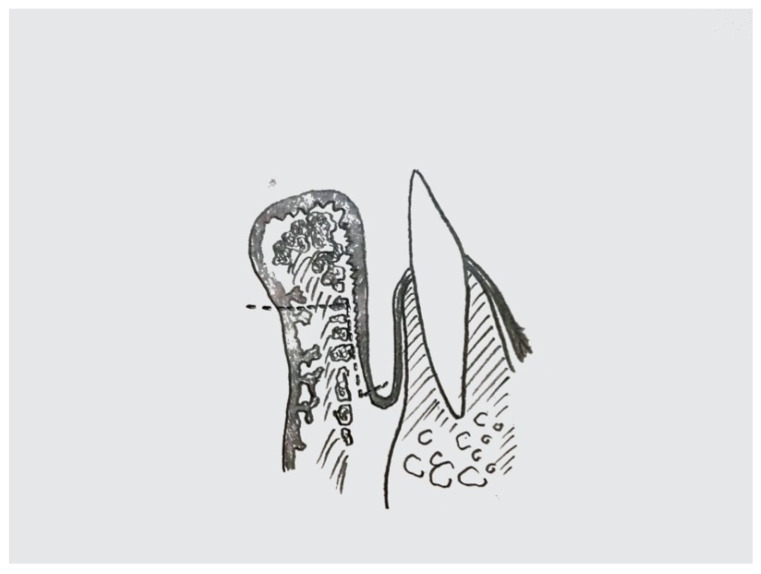
Vermillion sliding flap: incision at cross-section view.

**Figure A3:**
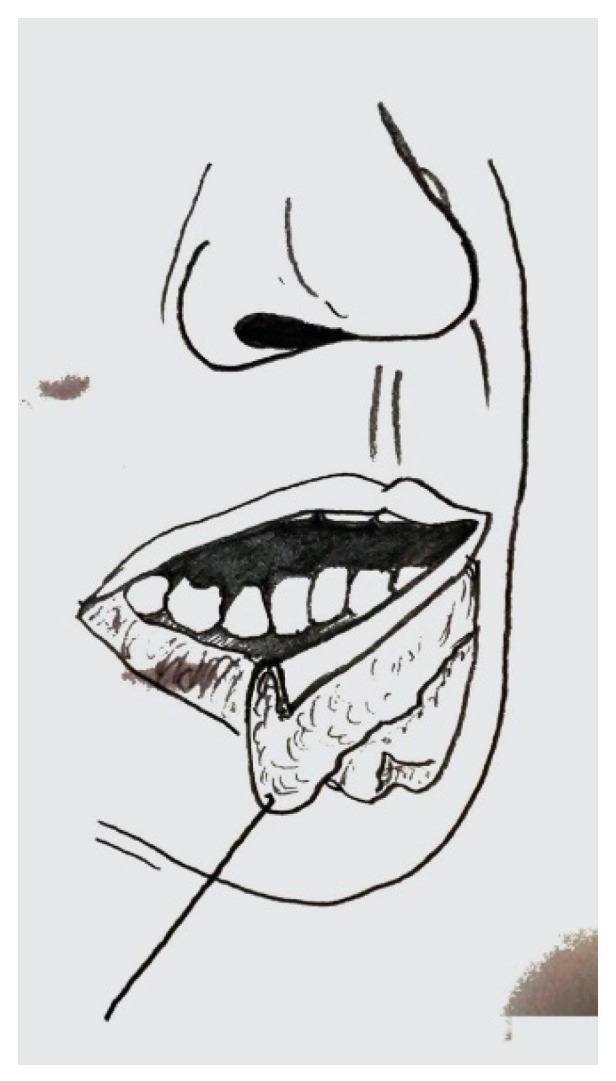
Vermillion sliding flap: tissue traction and positioning after sculpturing.

Flaps sculpture started with a cutaneous incision at the edge between the vermilion and the skin area, followed by a mucosa incision at the corresponding inner side of the lip (see Figure A2). Flap incision started 0.5 cm from the commissure of the healthy side to preserve the orbicularis vessels (see Figure A3). Because of the high elastic inherent characteristics of the muscular and vessel element of the flap, the extension was possible up to complete lip reconstruction for both upper and lower lip. In reconstructions involving the upper, lower lip, and modiolus, the two elastic flaps were sutured at the neo-modiolus site obtained from the suture of the upper and lower bundle of the contralateral buccinator muscle. The opposite tension of the two flaps was spontaneously balanced; thus, traction stitches were not necessary at the reconstructed side to fix the modiolus position.

## 3. Results

Results are summarized in Table 1 according to the following criteria: postoperative problems, the sensitivity of the lips with 2-point discrimination test (normal, correct, poor, protective, hypoesthesia), oral competence (poor, acceptable, good, excellent), microstomia (absent, mild, relevant), aesthetic results in movement (poor, acceptable, good, excellent), symmetry of the lips (poor, acceptable, good, excellent) concerning the evaluation of the doctor and patient satisfaction.

- Discrimination test (2PTD): normal (less than 6 mm), fair (6 to 10 mm), poor (11 to 15 mm), protective (one point perceived), hypoesthesia (no points perceived).
- Oral competence: excellent (normal situation), very good (good competence of lip with oral sphincter good continence), good (good competence of lip with acceptable oral sphincter continence), acceptable (acceptable competence of lip with poor oral sphincter continence), poor (poor competence of lip with poor oral sphincter continence).
- Microstomia: absent (a normal-like condition with normal deglutition and prosthesis insertion), mild (sufficient deglutition and prosthesis insertion), heavy (poor deglutition and no prosthesis insertion).
- Dynamic aesthetic results: excellent (normal situation), very good (very good aesthetic of lips with oral sphincter, very good aesthetic), good (good aesthetic of lips with acceptable oral sphincter aesthetic), acceptable (acceptable aesthetic of lips with poor oral sphincter aesthetic), poor (poor aesthetic of lips with poor oral sphincter aesthetic).
- Symmetry: excellent (normal situation), very good (very good symmetry of lip), good (good symmetry of lip), acceptable (acceptable symmetry of lips), poor (poor symmetry of lips).

In all cases, the follow-up visits assessed oral competence, commissure symmetry, the function of the sphincter system of the lips at rest and at movement during chewing and conversation, and the ability to wear the dental prosthesis.

In general, patients had an optimal postoperative course without any complications. The lesions were removed, preserving a safety margin of one centimeter from the lesion. The postoperative histological examination confirmed squamous cell carcinoma and PA with free margins in all cases. The postoperative staging was confirmed to be T1N0 for all patients. No ischemic complications have been detected in any of the patients.

Patients were evaluated in follow-up for dynamic symmetry and resting symmetry, oral competence, mouth opening, and the ability to wear the prosthesis. Restoration of lip sensitivity was assessed by a two-point discrimination test (2PDT) and overall patient satisfaction.

**Figure 1:**
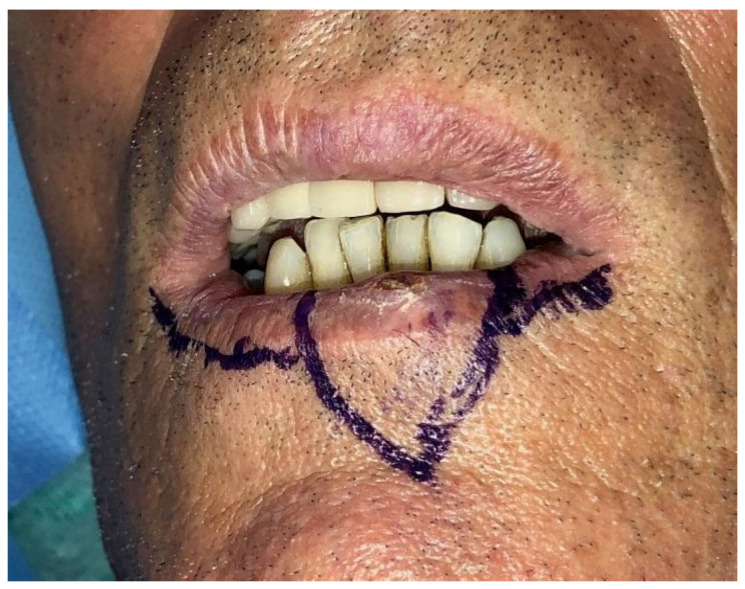
Case 1: Defect involving more than 1/3 of lower lip and the paramedian site.

**Figure 2:**
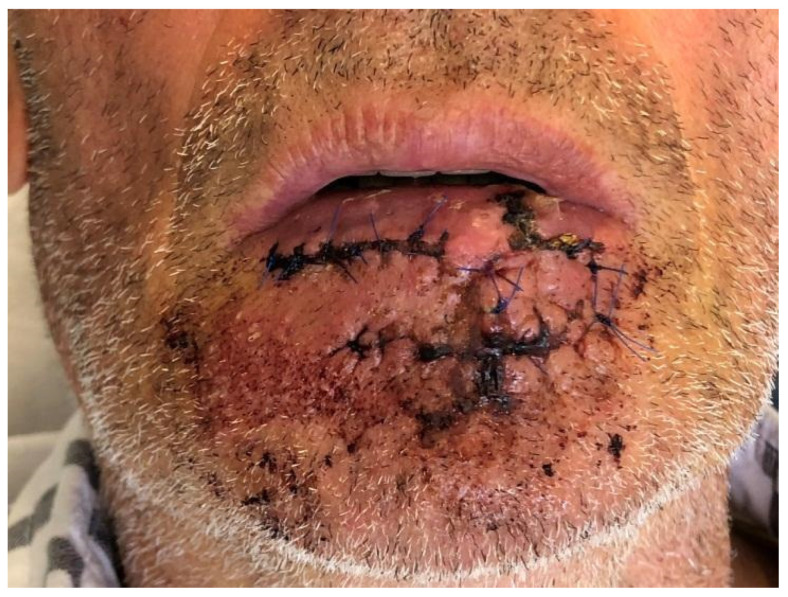
Case 1: Postoperative view.

**Figure 3:**
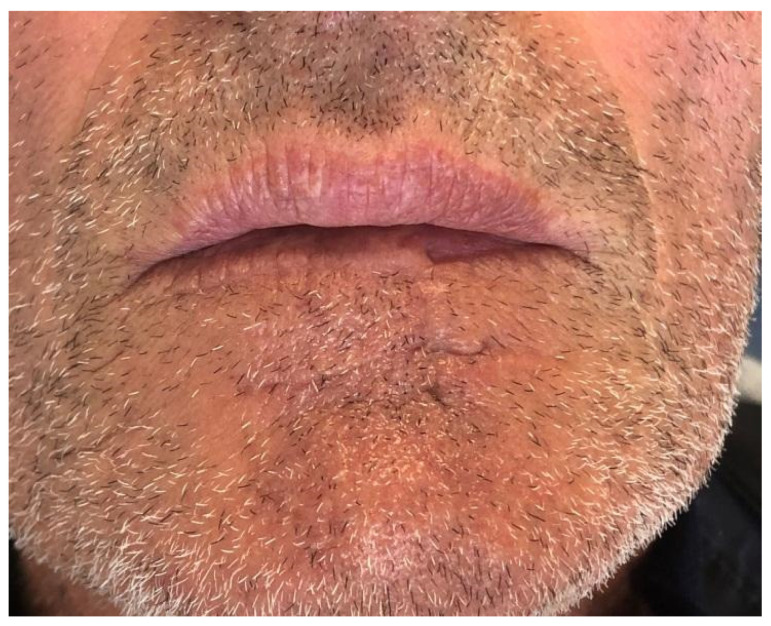
Case 1: Follow up at sixth months. Resting position.

**Figure 4:**
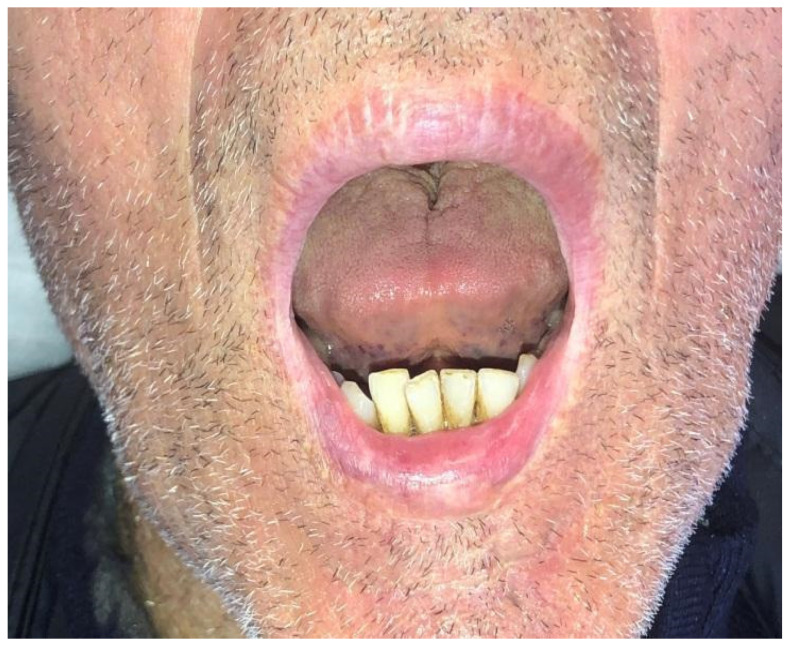
Case 1: Follow up at sixth months. Dynamic position.

Case 1 was a 67-years-old man in relatively good general health affected by squamous cell carcinoma, stage T1N0M0. This case, after ablative surgery, showed a lower lip defect involving more than 1/3 of the lower lip at the paramedian site (Figure 1). To reconstruct this defect, we modified the technique performing our double layer reconstructive technique using only the lower bundle of the buccinator muscle in association with a double sliding orbicularis muscle flap at both lateral sides of the lower lip. After resection, an incision was performed at the cutaneous edge of the vermilion and the cutaneous skin on both lateral aspects of the unaffected portion of the lower lip. The same incision was performed at the mucosa lip aspect, thereby elevating orbicularis and vermilion sliding flaps bilaterally. Sculpture of the buccinator flap dissected the modiolus and lower bundle of the buccinator muscle by a subcutaneous blunt dissection in the cheek, with only a mucosa incision to avoid skin scars obtaining the advancement of the composite muco-myo-cutaneous lower buccinator bundle flap. A portion of the cheek skin was transposed to reconstruct the lower lip cutaneous area at the lateral buccinator flap base. The same procedure was performed on the opposite side, allowing reconstruction of the cutaneous portion of the lower lip followed by reconstruction of the vermilion and orbicularis portion by sliding Robotti flap. Neo modiolus positioning was obtained suturing the upper bundle of the buccinator and the elongated lower bundle of the buccinator (Figure 2). Blunt dissection allowed the preservation of vessels and most sensitive and motor nerve branches, achieving good aesthetic and functional results (Figure 3 and Figure 4).

**Figure 5:**
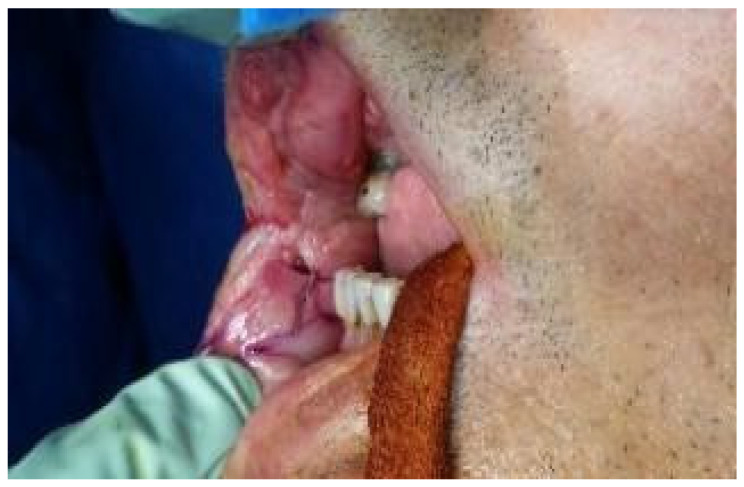
Case 2: Squamous cell carcinoma of the right commissure extending to the upper and lower lips.

**Figure 6:**
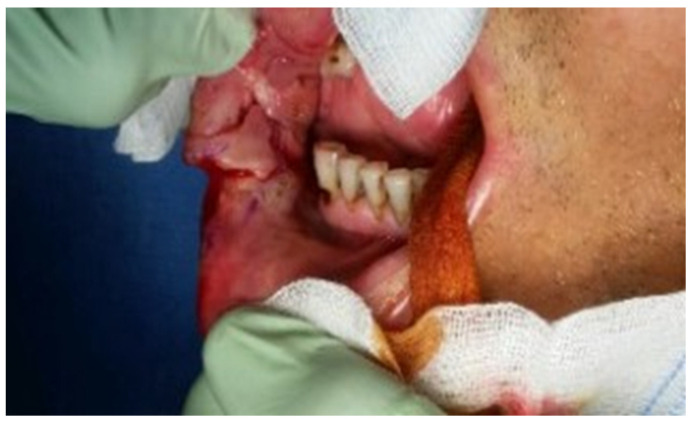
Case 2: Other view of the squamous cell carcinoma of the right commissure extending to the upper and lower lips.

**Figure 7:**
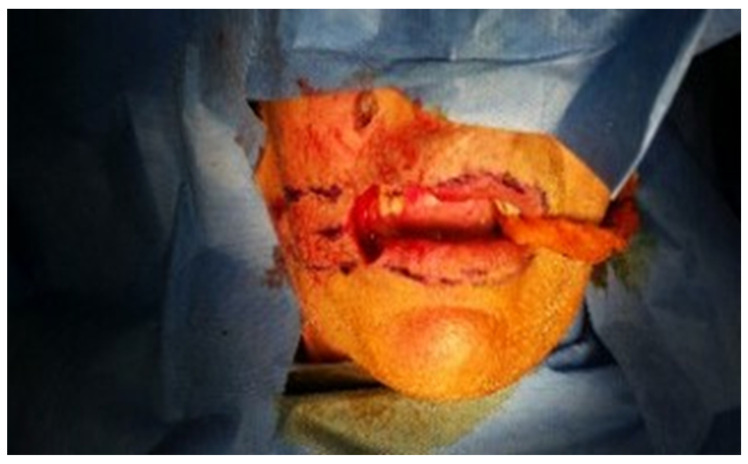
Case 2: excision and reconstructive planning.

**Figure 8:**
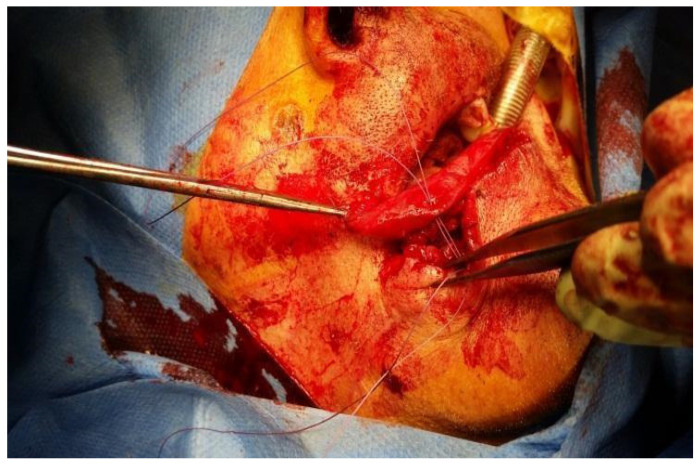
Surgical view of reconstruction following the new technique.

Case 2 was a 63-year-old man, in reasonably good general health, affected by squamous cell carcinoma of the right commissure extending to upper and lower lips, stage T1N0M0 (Figure 5 and Figure 6). The patient was treated with ablative surgery resulting in a defect involving the right commissure, 45% of the upper lip, to 25% of the lower lip, that was reconstructed with local flaps in the same surgical stage. The reconstruction was performed using the aforementioned association of two different flaps to restore both the cutaneous and vermillion structures of the lip by using a buccinator and an orbicularis sliding flap, respectively. A buccinator V-Y advancement muco-myo-cutaneous flap was used, modified by splitting the two bundles of the buccinator muscle, following the functional anatomy of the cheek-lip complex, where the fibers of the two bundles of the buccinator are connected to the two bundles of the upper and lower lip orbicularis oris muscle after modiolus insertion. In this way, the upper bundle was used to reconstruct the upper lip for the cutaneous area, and the lower beam was used to restore the lower lip skin area after resection. The modiolus was created by suturing the two buccinator bundles at the original position. The vermilion was reconstructed in association with this technique, combining two vermillion-orbicularis modified contralateral myo-mucosal flaps following the Goldstein–Robotti techniques (Figure 7 and Figure 8) [15].

**Figure 9:**
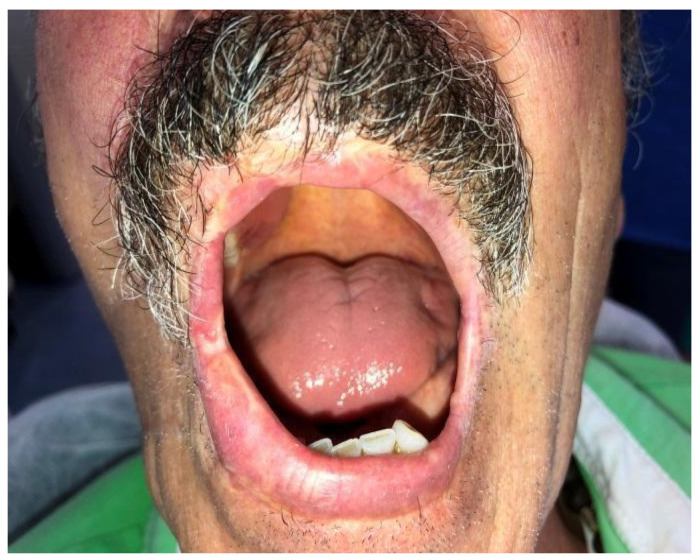
Case 2: Follow up at six months. Dynamic position, frontal view.

**Figure 10:**
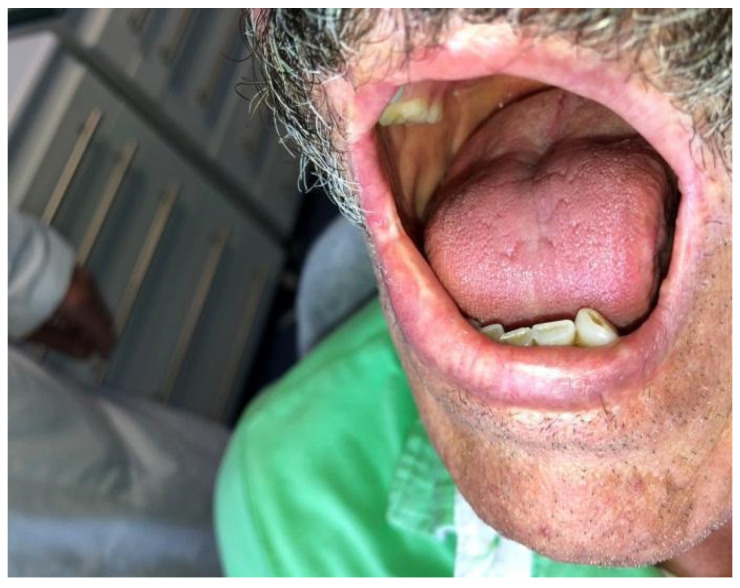
Case 2: Follow up at six months. Dynamic position, lateral view.

Due to the distinctly elastic properties of the orbicularis muscle and vessels, it was possible to reconstruct up to one-half of the lip by pulling the orbicularis muscle fibers from the healthy side towards the demolished side. This technique successfully reconstructed the resected lip without reducing the opposite (upper/lower) unaffected lip, thus avoiding a reduction of the orbicularis oris ring and lip skin area with a subsequent microstomia. Gaining tissue from the composite muco-myo-cutaneous flap of the cheek by careful blunt dissection, the buccinator and the skin portion preserved their vascularization and most of the innervation: transferring it to the demolished region. In combination with the Robotti technique, the lip was reconstructed in its two main components: the basal cutaneous and the vermillion areas, preserving most of the original sensitivity, motion, and symmetry (Figure 9 and Figure 10).

Case 3 was a 73-old-male patient affected by a squamous cell carcinoma involving the left commissure, stage T1, N0, M0. Resection was performed, resulting in a wide defect involving the left commissure, 40% of the lower-left lip, and 20% of the upper left lip. After reconstruction performed by the association of a buccinator full-thickness flap in association with a vermillion sliding flap contra-lateral to the defect, results were satisfactory without any postoperative problems. Two-point discrimination test, oral competence, aesthetic evaluation at motion and at rest were positive without any sign of relapse after 40 months follow-up.

Case 4 was a 60-years-old male heavy smoker affected by squamous cells carcinoma T1N0M0. Resection was performed, resulting in a wide defect involving 40% of the lower lip, the commissure, and 20% of the ipsilateral upper lip. After reconstruction, results were satisfactory, without any postoperative problems. Two-point discrimination test, oral competence, aesthetic evaluation at motion and at rest were positive without any sign of relapse after 24 months follow-up.

Case 5 was a 59-years-old male patient affected by a squamous cell carcinoma of the right commissure of the mouth extending to 25% of the upper lip. Resection and reconstruction were performed, and good results were obtained with good oral competence, good symmetry, and mouth opening without any signs of relapse after 38 months of follow-up.

**Figure 11:**
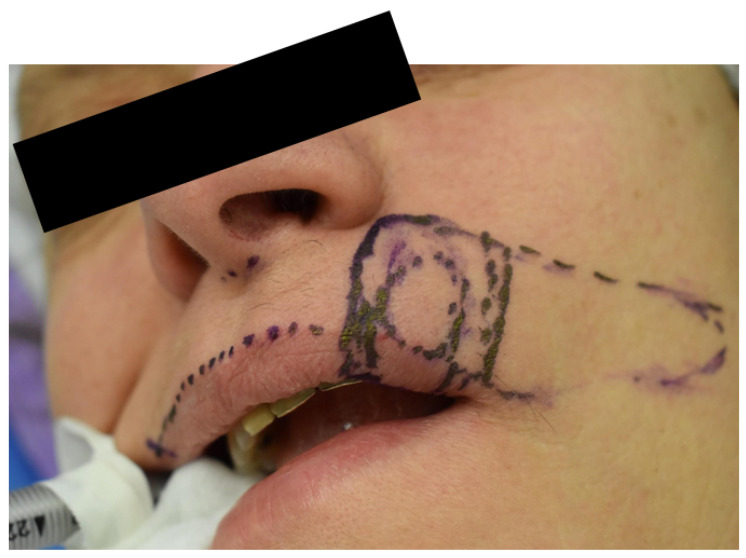
Case 6: Polymorphous low-grade adenocarcinoma of the left upper lip and reconstructive planning.

**Figure 12:**
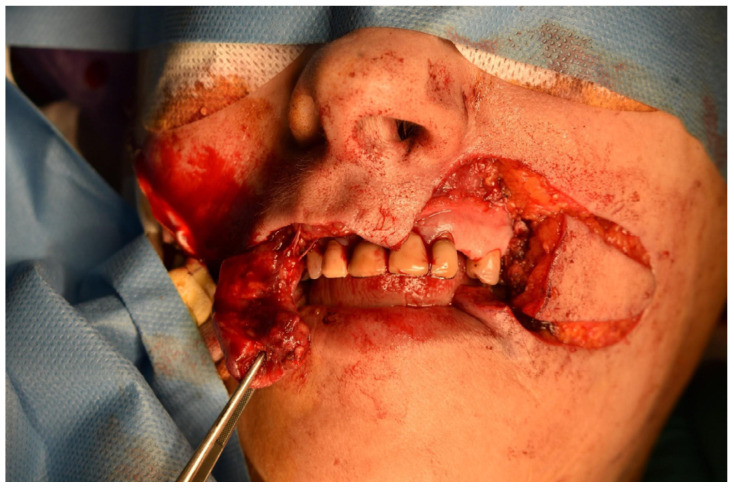
Defect involving 30% of the left upper lip and reconstruction by the new technique.

Case 6 was a 65-year-old female, in good general health, affected by polymorphous adenocarcinoma (PA) of the left upper lip, stage T1N0M0. The patient presented with a small asymptomatic swelling that resembled a benign tumor (Figure 11). A biopsy revealed the histological diagnosis of PA with a pushing pattern and infiltrative areas in the fat tissue, also showing perineural invasion. Pre-operative ct scan, echography, and intra-operative sentinel lymph node assessment revealed that the PA was only locally invasive; therefore, surgery alone was selected as a treatment. The surgical treatment was an ablative surgery of 30% of the left upper lip. A frozen histologic examination revealed that the lesion was well within 1 cm of free margins; furthermore, a negative sentinel lymph node was detected in the L1b station. After ablation, the defect reconstruction was performed by sculpturing a buccinator V-Y advancement muscle-muco-cutaneous flap (Figure 12).

**Figure 13:**
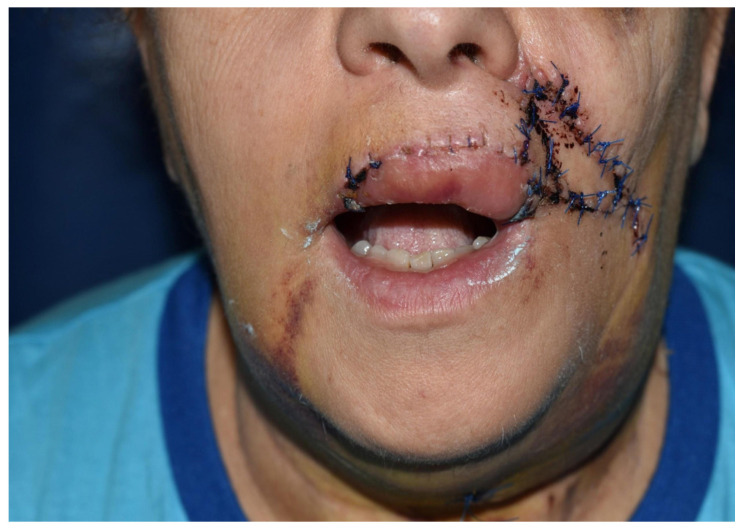
Case 6: Follow up at 8 days. Dinamic position, frontal view.

**Figure 14:**
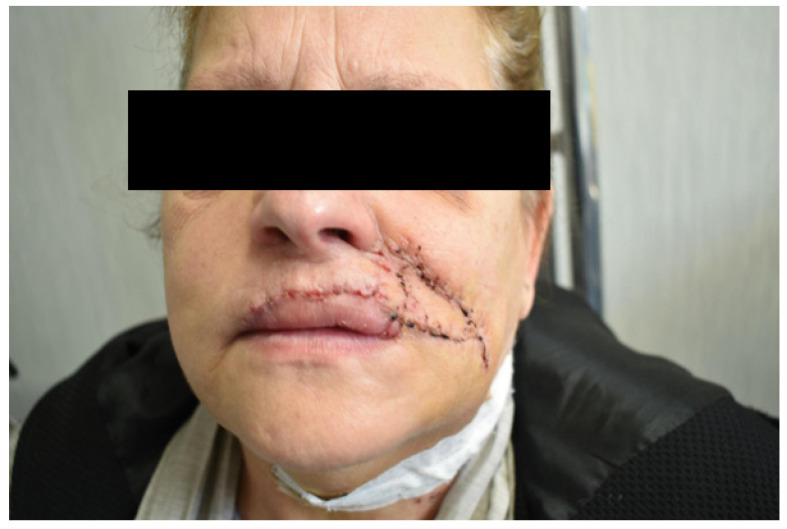
Case 6: Follow up at 15 days. Static position, frontal view.

**Figure 15:**
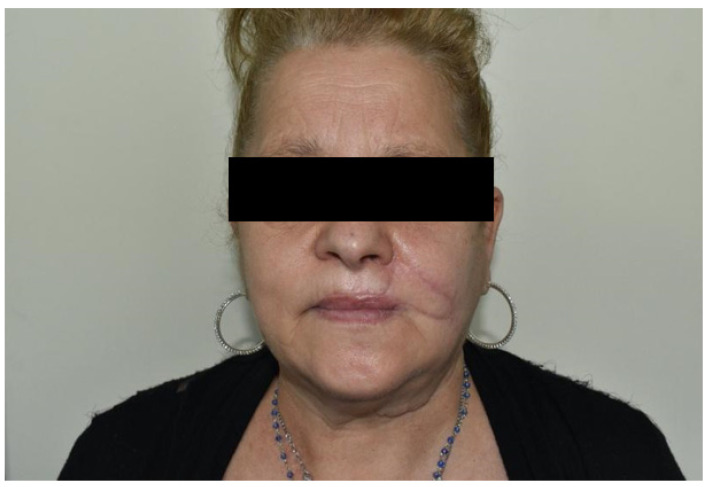
Case 6: Follow up at 6 months after surgery. Static position, frontal view.

**Figure 16:**
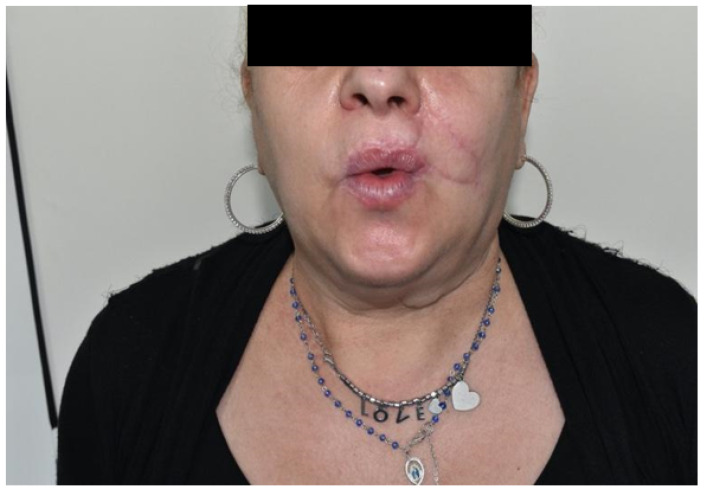
Case 6: Follow up at 6 months after surgery. Sphincteric muscle function, frontal view.

It was performed by an incision on both the cheek’s skin and inner mucosa aspects, with blunt dissection of the two buccinator muscle bundles preserving facial vessels and innervation. The neo-modiolus was reconstructed by suturing the upper buccinator bundle with skin and mucosa layers on the orbicularis muscle. The vermillion and orbicularis oris muscle rings were reconstructed using the elastic sliding flap following the Goldstein-Robotti technique. Good aesthetic and functional results were achieved: part of the sensitivity and motor innervation and the vascular supply was preserved, with no suture dehiscence or necrosis areas in the postoperative time. No microstomia was observed after surgery (Figure 13, Figure 14, Figure 15 and Figure 16).

## 4. Discussion

Following the basic concept of reconstructing “like with like” from one of the masters of plastic surgery, Dr. Gillies, we tried to reconstruct the challenging area of the lip with tissue flaps as similarly as possible to the resected ones by following the original technique of the buccinator flap in combination with the orbicularis and vermillion flap by Goldstein and Robotti [9].

Following the principles that a flap reconstruction is not successful if not symmetric, replacing the same shape, tissue texture, color as original and possibly innervated, by our technique, we tried to challenge the idea of Bakamjian that defined in 1964 this surgical endeavor as an “almost unreachable objective” because of its complexity [16].

The rationale of our procedure was based on the anatomical and functional observations that are discussed below.

Observing lip anatomy and function, we divided the lips into (1) cutaneous and (2) vermillion. These two lip areas are very different for structure (the skin area is less elastic and thicker, and the mucosa area is very elastic and delicate) and for their function with different mobility and appearance in smiling. To respect the principle to replace “like with like,” we decided to reconstruct the two different levels of the lips with two different flaps: a cutaneous one from the cheek for the skin portion and a vermillion one with mucosa for the same area from the opposite side of the same lip.

To preserve the anatomy and symmetry of the unaffected areas of the lips, in each reported case, we adopted the two different flaps based on opposite sides to balance tractions. Combining the cutaneous lip reconstruction using a buccinator flap from the ipsilateral cheek with a contralateral orbicularis muscle sliding flap for vermillion with sphincter function, the reconstruction of the proper anatomy, aesthetics, and function of the resected area was achieved in one surgical step. The aesthetic and symmetry of unaffected lip areas and the nose were also preserved, differently from many other most common techniques.

Another important aspect is the possibility of reconstructing both lips, including the modiolus, in one step. Looking at the anatomy of the buccinator muscle with its two bundles, evidence will support the natural continuation of the buccinator with the orbicularis after modiolus decussation. Based either on the facial artery or the buccal artery from the internal maxillary artery, use of this flap is also possible in case of facial artery ligation in neck dissection [17]. Advancement of a portion of buccinator bundles was possible after blunt dissection and V-Y skin and mucosa incisions.

Moreover, scar tissue was limited because of the blunt dissection was more conservative than the full thickness sections by common reconstructive techniques from opposite lips. Finally, the vermillion and orbicularis portion will result very similar to the resected ones because they gained from the remaining healthy portion of the lip and therefore matched to the original for weaving, structure, and color.

The functional and aesthetic evaluation of the reconstructed cases was satisfactory because of symmetry at rest and at motion when smiling, speaking, and chewing, for shape, annexes, tissue texture, and even for some sensitive and motor nerve recovery. Symmetry was also preserved at the unaffected lip areas of opposite and contralateral lip differently from other most common reconstruction techniques rotating flap from unaffected lip areas, with consequent tractions and asymmetry for the nose and other lip areas [15,18,19,20].

Aesthetic and functional results were considered for symmetry, shape, similarity in tissue texture, annexes, mouth opening, ability to chew and wear a prosthesis when necessary, and even for some neurosensory preservation or recovery, as shown in Table 1. Other authors using similar techniques (without similar aesthetic and functional results) reported neurosensory preservation or recovery after reconstruction, confirming the technique’s rationale [20].

Similar results concerning neurosensory preservation were shown and discussed in the literature by other authors adopting similar techniques (modified Bernard-Webster technique) [20]. Their results are quite unsatisfactory for aesthetic and function because of lack in vermillion reconstruction and microstomia resulting from the use of tissue from the unaffected upper lip, unlike the cases reconstructed with our technique.

Another article reported a similar new technique that combines a vermillion myomucosa flap with the Abbe–Estlander flap showed evident microstomia after using tissue from the unaffected upper lip [15], differently from our cases showing absent microstomia and respected alar nose symmetry.

It was also possible by our technique to reconstruct more extensive resections involving modiolus and upper and lower lip or only a single portion of the lip for limited resections. Transposition of the tissue from the cheek had the substantial advantages of avoiding tractions on the nose or unaffected lip areas without evident microstomia compared to the other methods that reconstruct the demolished lip by taking tissue from the opposite lip thereby reducing the size of the orbicularis ring.

In our opinion, to reconstruct both components of the lips, the most suitable solution is the association of the buccinator muscle flap for the less elastic cutaneous lip area, with the vermillion and orbicularis sliding flap, which can reconstruct up to half of the lip without any substantial reduction in the oral sphincter.

## 5. Conclusions

In conclusion, the results obtained in our case series point out the effectiveness of our technique as a fulfilling solution to the basic principles of flap reconstruction, such as the “like to like” concept and the preservation of neurosensory and vascular supply and both static and dynamic symmetry of the lips. These conditions are not completely observed altogether in most current techniques.

Our technique replaced the integrity of the orbicularis oris by pulling the vermillion of the contralateral portion of the lip towards the resected side, thereby reconstructing the vermillion thanks to the wide elasticity of this part of the lip (as evidenced in smile movements).

The cutaneous portion of the resected lip in our technique was reconstructed, gaining tissue from the check structures, splitting the two bundles of the buccinator muscle.

Based on the facial artery or on the buccal artery from the maxillary artery (Zhao) (useful option in case of facial artery ligation after neck dissection), advancement of a portion of buccinator bundles was performed after blunt dissection and V-Y skin and mucosa incisions [17].

Because of combined contralateral flaps reconstruction, lip symmetry was also preserved together with function due to the double layer lip reconstruction preserving different anatomy and functions of the cutaneous and vermillion lip portions. Aesthetic symmetry of the unaffected lip and nose were preserved by avoiding flap raise from the opposite lip.

Although the results observed using our new technique are satisfactory, a larger number of cases will be needed to validate the technique.

## Figures and Tables

**Table 1:** Patients’ characteristics summary.

Patient No.	Sex	Age (Years)	Diagnosis	Defect Site	Post-Operative Problems	2PDT	Oral Competence	Microstomia	Dynamic Aesthetic Results	Symmetry	Follow-up (Months)
1	M	67	SCC	Up to 30% of middle portion of the lower lip	None	Protective	Very good	Absent	Very good	Very good	6
2	M	63	SCC	R commissure extending to 45% of the upper lip and 25% of the lower lip	None	Protective	Very good	Absent	Very good	Very good	35
3	M	73	SCC	L commissure and 40% of the lower L lip and 20% of the upper L lip	None	Protective	Good	Absent	Good	Good	40
4	M	60	SCC	40% of the lower lip commissure and 20% of the ipsilateral upper lip	None	Protective	Good	Absent	Good	Good	24
5	M	59	SCC	R commissure extending to 25% of the upper lip	None	Protective	Good	Absent	Good	Good	38
6	F	65	PA	30% of the left upper lip	None	Protective	Very good	Absent	Good	Very good	1

M: male; F: female; SCC: squamous cell carcinoma; PLGA: polymorphous adenocarcinoma; 2PDT: two-point discrimination test.

## Data Availability

Data is available upon reasonable request.
